# Supplementary material for: Parent and Child Choice of Sugary Drinks Under Four Labelling Conditions
Source: Nutrients. 2025 Jun 3;17(11):1920. doi: 10.3390/nu17111920 (PMC12157589; doi:10.3390/nu17111920)
Supplement: Supplementary file 1 [file nutrients-17-01920-s001.zip › nutrients-3622282-supplementary.pdf]

Figure S1.

Vending machine image, teaspoons of sugar condition

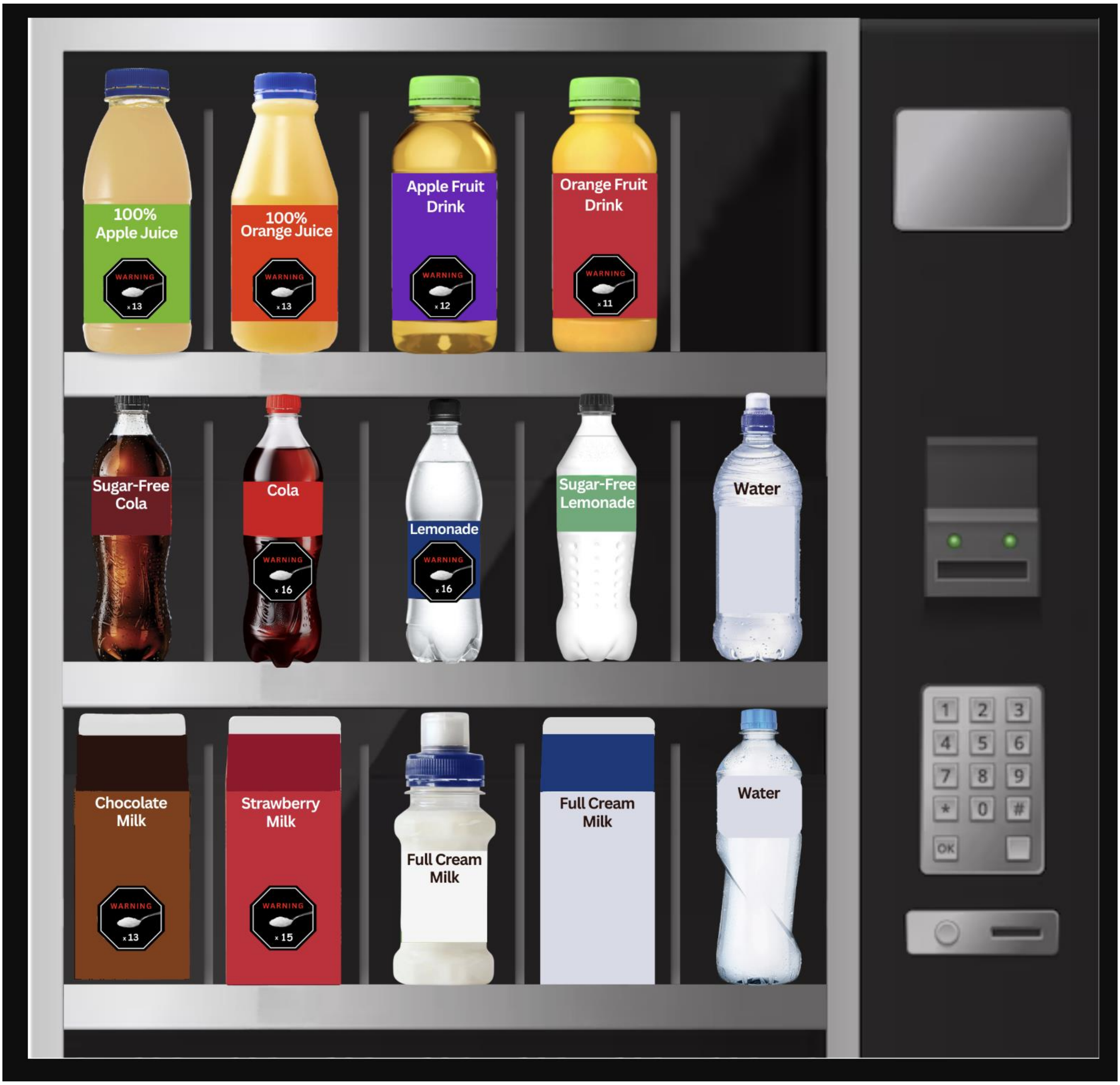

Figure S2.

Vending machine image, tooth decay pictorial condition

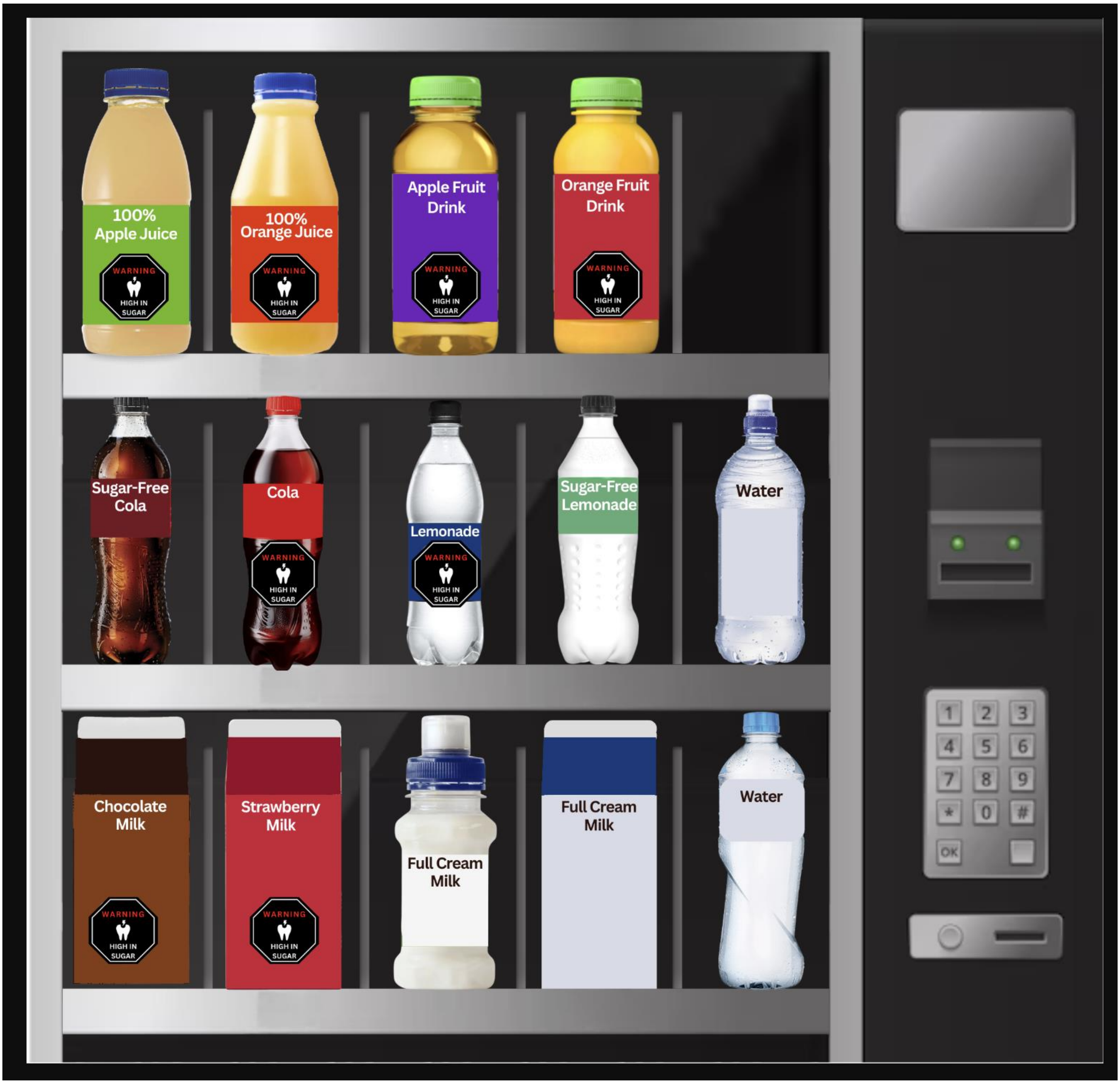

Figure S3.

Vending machine image, text-based health warning condition

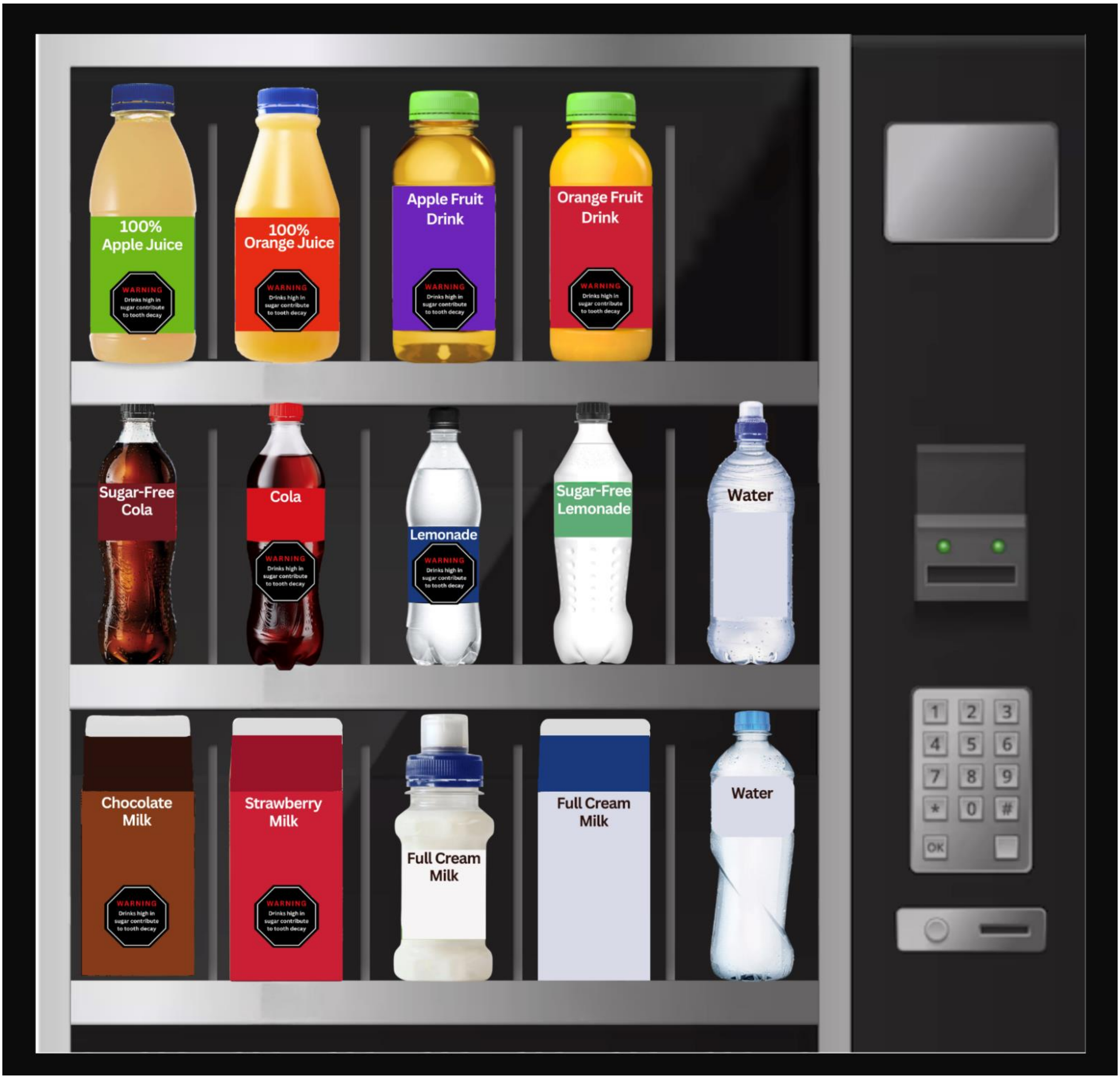

Figure S4.

Vending machine image, HSR condition

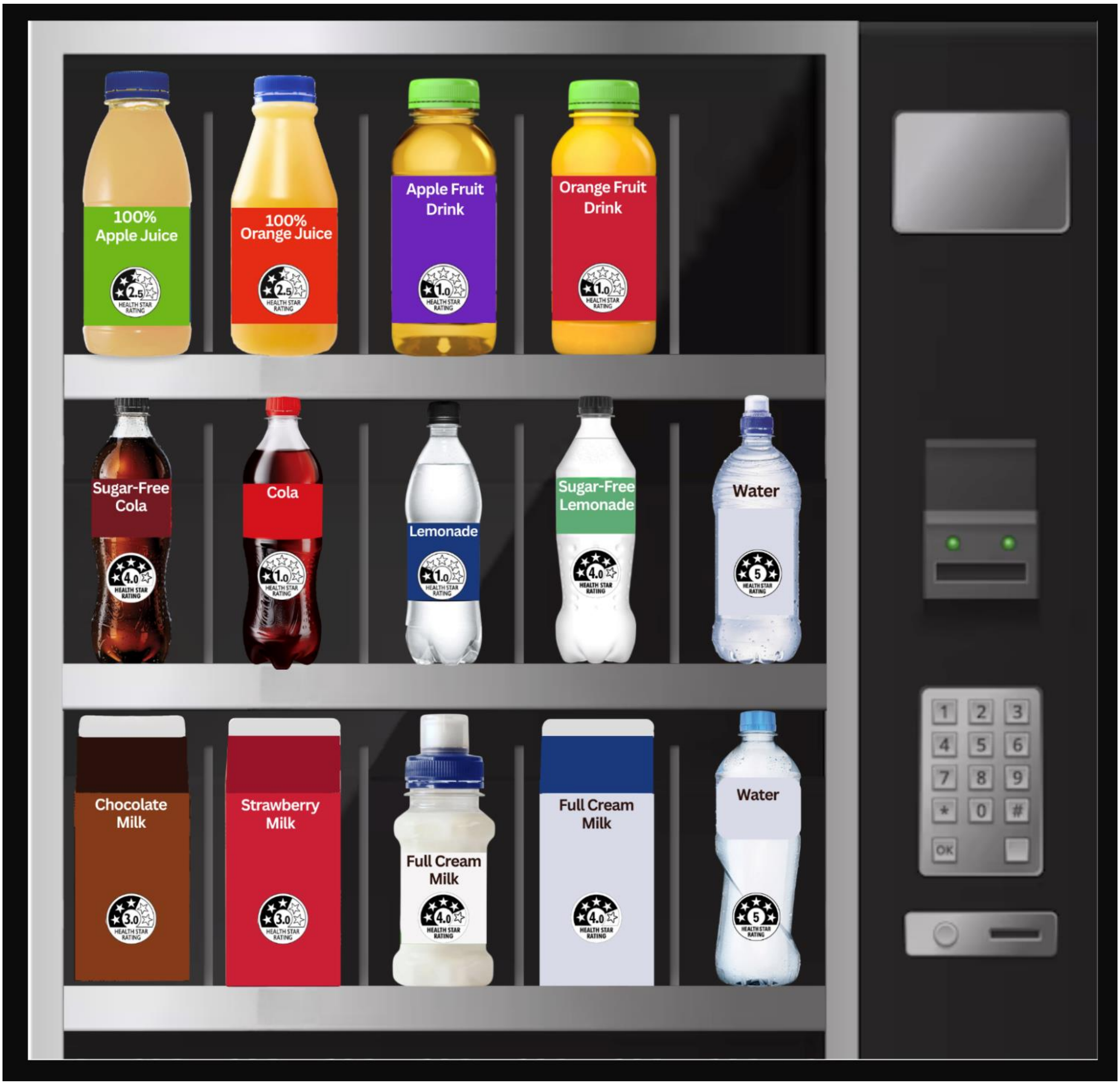

Table S1.

Beverage Healthiness Rankings

Drink Category	Final Ranking	Rating Scores		
		Range	Mean	Median
Tap Water	1	9-10	9.9	10
Full Cream Milk	2	5-10	8.0	8
100% Juice	3	2-8	5.3	6
Flavoured Milk	3	2-8	5.0	5
Fruit Drink	4	1-5	2.9	3
NNS	4	1-5	2.6	3
Soft Drink	5	1-2	1.2	1
